# Multi-Tissue Transcriptomic Analysis Reveals Tissue-Specific Thermal Responses and Sex-Biased Functional Differentiation in Reproductive Tissues of the Brown Frog (*Rana dybowskii*)

**DOI:** 10.3390/cimb48060583

**Published:** 2026-06-01

**Authors:** Shifang Zhang, Guo Hu, Yuan Guo, Xudong Chang, Haijun Li, Yingzhu Liu

**Affiliations:** 1 Institute of Natural Resources and Ecology, Heilongjiang Academy of Sciences, Harbin 150040, China; zhangshifang2022@126.com (S.Z.); guoyuan010189@126.com (Y.G.);; 2 Heilongjiang River Fisheries Research Institute, Chinese Academy of Fishery Sciences, Harbin 150070, China

**Keywords:** brown frog (*Rana dybowskii*), transcriptome, temperature response, tissue specificity, reproductive tissues

## Abstract

The brown frog (*Rana dybowskii*) is an important cold-temperate amphibian species in northeastern China, with considerable ecological and resource value. Because its survival, metabolism, and reproduction are highly sensitive to environmental temperature, elucidating the molecular mechanism’s underlying temperature response is of great significance for understanding environmental adaptation and reproductive regulation in amphibians. In this study, *R. dybowskii* was used as the experimental model, and a multi-tissue transcriptomic analysis was conducted on the brain, liver, spleen, ovary, oviduct, and testis from both females and males under 2 °C and 12 °C conditions to characterize tissue- and sex-specific responses to temperature variation. The results showed that the global transcriptomic landscape of *R. dybowskii* was primarily driven by tissue type, whereas temperature effects were mainly manifested within individual tissues. The liver, brain, and spleen exhibited pronounced temperature responsiveness in both sexes, with the liver mainly showing metabolic reprogramming-related functional changes. Intersection analysis further revealed that temperature-responsive genes included not only conserved modules shared across tissues but also a large number of tissue-specific genes. Reproductive tissues displayed more pronounced functional divergence: the ovary showed relatively limited transcriptional changes and remained comparatively stable; the oviduct underwent marked transcriptional remodeling, with upregulated genes mainly involved in glycosylation and macromolecule processing, whereas downregulated genes were primarily associated with ribosomes, translation, and RNA splicing; the testis was likewise highly sensitive to temperature changes, with upregulated genes mainly enriched in amino acid metabolism, mitochondrial function, and DNA repair, while downregulated genes were mainly related to cellular structure and intercellular junctions. Collectively, these findings indicate that the molecular adaptation of *R. dybowskii* to temperature variation is characterized by strong tissue specificity and sex-biased responses in reproductive tissues. This study provides a transcriptomic basis for elucidating the molecular responses to short-term temperature variation is important for understanding environmental sensitivity and reproductive regulation in amphibians.

## 1. Introduction

Temperature is a key environmental factor affecting the survival, metabolism, immunity, and reproduction of amphibians. As typical ectotherms, amphibians are highly dependent on external thermal conditions and are therefore generally considered particularly vulnerable to climate warming and temperature fluctuations. Recent studies have shown that rising temperatures and extreme thermal events can alter the thermal ecology and physiological performance of amphibians, and may further compromise individual health and fitness by affecting stress responses and immune defense [1]. In addition, climate change-induced shifts in temperature and precipitation can influence reproductive phenology and breeding success in amphibians, suggesting that thermal stress is associated not only with individual survival but also with the maintenance and renewal of populations [2,3].

Temperature-induced molecular responses are usually not mediated by a single organ, but instead involve coordinated regulation among multiple tissues and organ systems. Previous transcriptomic studies have shown that temperature changes can induce marked, yet not entirely consistent, gene expression reprogramming across different tissues, with the underlying processes commonly involving mitochondrial function, lipid and carbohydrate metabolism, antioxidant responses, apoptosis, RNA processing, and protein degradation [4,5]. Therefore, tissue specificity is an important dimension for understanding how ectothermic vertebrates respond to short-term temperature variation. RNA-seq has proven to be an effective tool for elucidating tissue-specific expression patterns and environmental adaptation processes in non-model amphibians, although multi-tissue systematic studies in such species remain relatively limited [6]. Compared with somatic tissues, reproductive tissues are often more sensitive to temperature fluctuation because gametogenesis, hormone regulation, gamete maturation, and reproductive tract homeostasis can all be affected by thermal conditions [7,8]. Moreover, temperature responses may differ not only among tissues but also between sexes. Some reproductive tissues may respond rapidly to environmental changes through extensive transcriptional remodeling, whereas others may maintain a relatively stable molecular state to protect key reproductive processes [9,10]. Thus, examining temperature responses from both tissue-specific and sex-specific perspectives is essential for understanding the balance between environmental response and reproductive maintenance in amphibians.

*Rana dybowskii* is a representative cold-temperate amphibian widely distributed in northeastern China and experiences marked seasonal temperature variation. Because this species undergoes long-term overwintering and subsequent spring warming in its natural habitat, it provides a suitable model for investigating short-term transcriptomic responses to temperature change [11]. Previous studies on *R. dybowskii* have mainly focused on phenotypic traits, physiological indices, reproductive biology, or individual tissues [12]. However, how different tissues and sexes coordinately respond to short-term temperature exposure at the transcriptomic level remains unclear [13,14].

The oviduct of female *R. dybowskii* is a reproductive tissue with marked seasonal physiological changes and is also associated with the formation of Oviductus Ranae. In the present study, we reduced the emphasis on the commercial value of Oviductus Ranae and focused instead on the biological relevance of the oviduct as a temperature-sensitive reproductive tissue. Previous studies have shown that the oviduct undergoes substantial molecular and functional changes during different physiological stages, suggesting that this tissue may possess considerable transcriptional plasticity and environmental responsiveness [11,15]. However, whether the oviduct, ovary, and testis exhibit distinct temperature-response patterns, and whether these responses differ between females and males, remains to be clarified.

Against this background, the present study used *R. dybowskii* as the study species and constructed a multi-factor transcriptomic dataset incorporating temperature, sex, and tissue. Brain, liver, spleen, ovary, oviduct, and testis tissues were collected from female and male individuals exposed to 2 °C and 12 °C. Here, the 2 °C condition was regarded as a low-temperature hibernation-related condition, whereas the 12 °C condition was used to represent short-term warming associated with emergence from hibernation, rather than long-term thermal adaptation. We hypothesized that short-term temperature exposure would induce tissue-specific and sex-biased transcriptomic responses in *R. dybowskii*, with somatic tissues and reproductive tissues displaying distinct functional response patterns. To test this hypothesis, we first evaluated global transcriptomic variation among tissues, sexes, and temperature treatments, then identified temperature-responsive differentially expressed genes within each tissue, and further analyzed shared and tissue-specific response modules. Finally, GO and KEGG enrichment analyses were used to characterize the major biological processes and pathways associated with short-term temperature responses in representative somatic and reproductive tissues. The purpose of this study was to clarify the tissue-specific and sex-biased transcriptomic responses of *R. dybowskii* to short-term temperature exposure, and to provide a molecular basis for understanding temperature-related physiological regulation and reproductive tissue differentiation in this cold-temperate amphibian.

## 2. Materials and Methods

### 2.1. Experimental Animals and Temperature Treatments

A total of 12 healthy sexually mature adult *R. dybowskii* individuals were used in this study. The frogs were collected from the Yangjiatun Aquaculture Experimental Station, Acheng District, Harbin, Heilongjiang Province, China. All individuals were adults with similar body condition, and sexual maturity was determined based on external morphological characteristics and further confirmed by gonadal development during dissection.

Considering that *R. dybowskii* is relatively sensitive to transport and environmental changes, all individuals were transferred to an indoor holding system for acclimation prior to formal treatment. Referring to the experimental design used in previously published multi-tissue transcriptomic studies of *R. dybowskii*, acclimation was conducted under dark, temperature-controlled, and shallow-water conditions. Briefly, frogs were maintained in tanks measuring approximately 80 cm × 35 cm × 40 cm, containing fully aerated distilled water at a depth of approximately 5 cm. The acclimation was conducted at 2 °C for 7 d under dark conditions. No food was provided during the acclimation period because the frogs remained in a hibernation-related state.

After acclimation, individuals were divided into female and male groups. Subsequently, individuals of each sex were randomly assigned to either the low-temperature group or the short-term warming group. For each sex × temperature combination, three biological replicates were included, corresponding to three individuals per group. Thus, the experimental design included three females at 2 °C, three females at 12 °C, three males at 2 °C, and three males at 12 °C [13].

The treatment temperatures were set at 2 °C and 12 °C for the low-temperature and short-term warming groups, respectively, and the treatment duration was 12 h. The 2 °C treatment was used to represent a low-temperature hibernation-related condition, whereas the 12 °C treatment was designed to simulate short-term warming associated with emergence from hibernation in early spring. These two temperatures were selected according to the seasonal biology of *R. dybowskii* in northeastern China, where overwintering individuals experience low environmental temperatures during hibernation and are exposed to increasing temperatures during spring emergence. Therefore, the 12 h exposure was intended to capture early transcriptomic responses to short-term temperature change rather than long-term thermal adaptation.

### 2.2. Tissue Collection and Transcriptome Sequencing

After completion of the temperature treatment, experimental individuals were rapidly dissected under RNase-free conditions for tissue collection. The double pithing technique, a standard humane euthanasia method for frogs, is applied for the disposal of individual experimental frogs. In females, brain, liver, spleen, ovary, and oviduct tissues were collected, whereas in males, brain, liver, spleen, and testis tissues were collected. All tissue samples were immediately frozen in liquid nitrogen and stored at −80 °C until RNA extraction.

Total RNA was extracted using TRIzol Reagent (Thermo Fisher Scientific, Waltham, MA, USA). RNA integrity and degradation were initially assessed by 1% agarose gel electrophoresis, while RNA concentration and purity were determined using a NanoDrop 2000 spectrophotometer (Agilent Technologies, Santa Clara, CA, USA). RNA integrity was further evaluated using an Agilent 2100 Bioanalyzer. Only high-quality RNA samples that met the requirements for library construction were used for subsequent sequencing analyses. For each sample, 3 μg of total RNA was used as the input material for library preparation, which was performed by the Experimental Department of BioMarker Technologies Corporation (Qingdao, China). The main procedures included mRNA enrichment or rRNA removal, RNA fragmentation, cDNA synthesis, end repair, adaptor ligation, and PCR amplification. After library construction, clustering was performed using the cBot Cluster Generation System, and paired-end sequencing was carried out on the Illumina NovaSeq 6000 (Illumina, San Diego, CA, USA) platform with a PE150 strategy. This sequencing platform and PE150 strategy were consistent with those used in the referenced study on *R. dybowskii* [13].

### 2.3. Sequencing Data Quality Control, Read Alignment, and Expression Quantification

Raw sequencing data were first subjected to quality control using Trimmomatic (v0.40) to remove adaptor-contaminated reads, low-quality reads, and reads containing a high proportion of ambiguous bases (N), thereby generating clean reads [16]. The filtered high-quality reads were then aligned to the *R. dybowskii* reference genome (https://www.ncbi.nlm.nih.gov/datasets/genome/GCF_905171775.1) (accessed on 23 April 2026) using HISAT2 (v2.1.0) [17]. The resulting BAM files were sorted and indexed for subsequent expression quantification. Based on the reference genome annotation file, gene expression levels in each sample were quantified using featureCounts (v2.1.1), and a raw read count matrix was generated [18].

### 2.4. Differential Expression Analysis

Differential expression analysis was performed in the R environment using the DESeq2 (v4.5.0) package for normalization and significance testing [19]. Given the substantial baseline expression differences among tissues and the pronounced sex specificity of reproductive tissues, a tissue-stratified analytical strategy was adopted in this study. Specifically, differential expression between the high-temperature and low-temperature groups was assessed separately within each tissue, while sex-specific tissues were analyzed independently within the corresponding sex.

DESeq2 was used to normalize the raw count data and test for differential expression based on a negative binomial distribution model. Genes with an adjusted significance level of padj < 0.05 and |log2FoldChange| ≥ 1 were defined as differentially expressed genes (DEGs). Among these, genes with log2FoldChange > 0 were considered upregulated under high-temperature treatment, whereas genes with log2FoldChange < 0 were considered downregulated under high-temperature treatment [20].

### 2.5. Functional Enrichment Analysis

To investigate the potential biological functions of temperature-responsive genes, GO (Gene Ontology) and KEGG (Kyoto Encyclopedia of Genes and Genomes) enrichment analyses were performed for differentially expressed genes using the R package (v4.5.0) clusterProfiler. Enrichment analysis was conducted separately for the DEGs identified in each comparison to detect significantly enriched biological processes, molecular functions, cellular components, and metabolic or signaling pathways [21].

Significantly enriched GO terms and KEGG pathways were identified using an adjusted *p* value threshold of <0.05. For DEGs shared among multiple tissues and for tissue-specific DEGs, functional annotation and enrichment analyses were further performed separately to distinguish conserved response mechanisms from tissue-specific regulatory patterns involved in temperature response in *R. dybowskii*.

All statistical analyses were conducted in the R environment. PCA plots, correlation heatmaps, DEG summary plots, volcano plots, and enrichment plots were generated using relevant R visualization packages, including ggplot2, patchwork, pheatmap, and visualization tools associated with clusterProfiler (v4.5.0) [22].

## 3. Results

### 3.1. Data Quality and Sample Clustering

**Figure 1:**
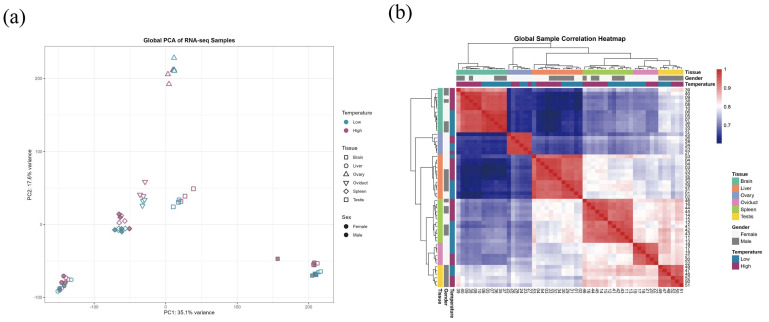
Global transcriptomic landscape of samples from different temperatures, sexes, and tissues in the brown frog (*R. dybowskii*). (**a**) Principal component analysis (PCA) based on the global gene expression matrix. Colors indicate temperature treatments, and shapes indicate tissue types. (**b**) Sample-to-sample correlation heatmap with hierarchical clustering. Annotation bars indicate tissue, sex, and temperature for each sample. The color scale from blue to red represents low to high correlation coefficients.

To evaluate the overall transcriptomic relationships among samples under different temperature, sex, and tissue conditions, principal component analysis (PCA) and sample correlation analysis were performed based on the global gene expression matrix. The PCA results showed that PC1 and PC2 explained 35.1% and 17.6% of the total variance, respectively (Figure 1a). Samples were separated into multiple clusters in the two-dimensional PCA space, and the clustering pattern was mainly associated with tissue type. Samples from the same tissue were generally grouped close to each other, whereas samples from different tissues were clearly separated. Within most tissue clusters, samples from the 2 °C and 12 °C groups were distributed relatively close to each other, and male and female samples did not show obvious separation in most somatic tissues.

The sample-to-sample correlation heatmap showed a similar clustering pattern (Figure 1b). Samples from the same tissue generally exhibited higher correlation coefficients, whereas correlations between different tissues were relatively lower. Hierarchical clustering further grouped the samples into several tissue-related clusters, consistent with the PCA results. These results showed that tissue type accounted for the major variation in the global transcriptomic dataset, while temperature- and sex-related differences were mainly observed within individual tissue groups. Therefore, subsequent differential expression analyses were performed separately within each tissue to reduce the influence of baseline expression differences among tissues.

### 3.2. Differential Expression Analysis Reveals Tissue Specificity

**Figure 2:**
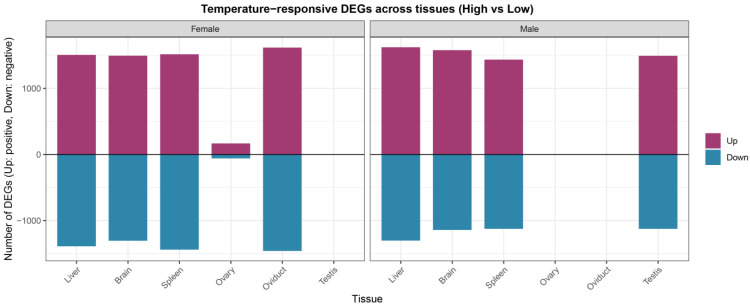
Distribution of the numbers of temperature-responsive differentially expressed genes (DEGs) across different tissues in female and male *R. dybowskii*. The numbers of upregulated and downregulated genes were calculated by comparing gene expression differences between the high-temperature and low-temperature groups within each tissue. Positive values indicate the numbers of upregulated genes, whereas negative values indicate the numbers of downregulated genes.

To compare the transcriptional responses of different tissues to short-term temperature exposure, differentially expressed genes (DEGs) were identified between the 12 °C and 2 °C groups within each tissue and sex, respectively. DEGs were defined using the criteria of adjusted *p* value < 0.05 and |log2FoldChange| ≥ 1. The numbers of upregulated and downregulated DEGs in each comparison are shown in Figure 2 and summarized in Table 1.

In females, DEGs were detected in all examined tissues. The liver contained 2896 DEGs, including 1507 upregulated and 1389 downregulated genes. The brain contained 2800 DEGs, with 1495 upregulated and 1305 downregulated genes. The spleen contained 2956 DEGs, including 1516 upregulated and 1440 downregulated genes. Among female reproductive tissues, the ovary showed the lowest number of DEGs, with 226 DEGs, including 167 upregulated and 59 downregulated genes. In contrast, the oviduct contained 3077 DEGs, including 1617 upregulated and 1460 downregulated genes.

In males, DEGs were also identified in all examined tissues. The liver contained 2926 DEGs, including 1623 upregulated and 1303 downregulated genes. The brain contained 2720 DEGs, with 1578 upregulated and 1142 downregulated genes. The spleen contained 2560 DEGs, including 1435 upregulated and 1125 downregulated genes. The testis contained 2618 DEGs, including 1493 upregulated and 1125 downregulated genes.

Overall, the number of DEGs varied among tissues and between sexes. In females, the oviduct had the highest number of DEGs, whereas the ovary had the lowest. In males, the liver showed the highest number of DEGs, followed by the brain, testis, and spleen. These results show that the transcriptional response to short-term temperature exposure differed among tissues and between sexes.

### 3.3. Overlap Analysis of Temperature-Responsive Genes Across Multiple Tissues

**Figure 3:**
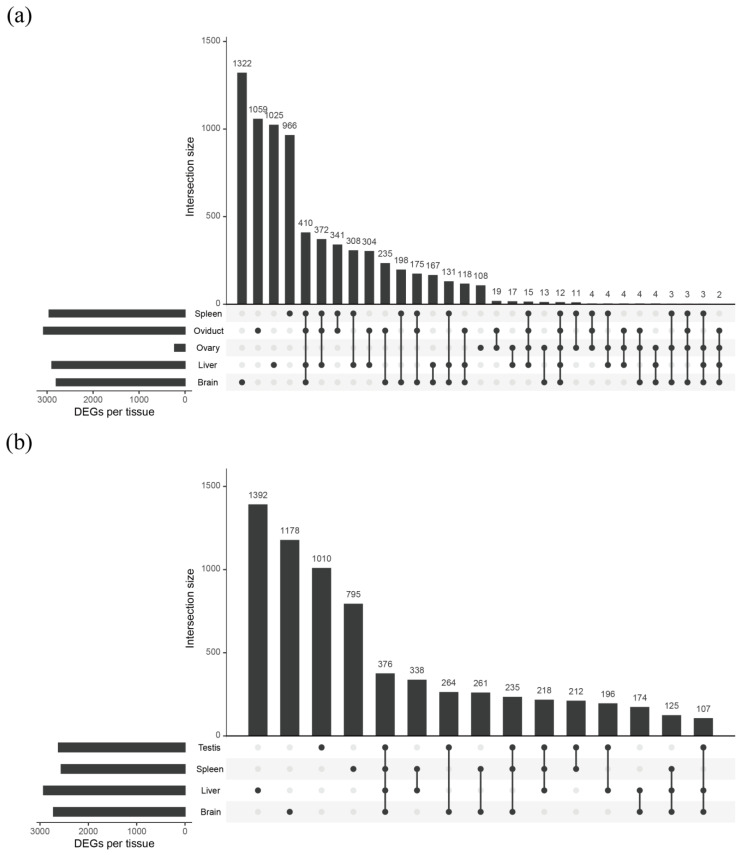
UpSet analysis of temperature-responsive differentially expressed genes (DEGs) across different tissues in female and male *R. dybowskii*. (**a**) Intersection patterns of DEGs among the brain, liver, ovary, oviduct, and spleen in females. (**b**) Intersection patterns of DEGs among the brain, liver, spleen, and testis in males.

To characterize the shared and tissue-specific patterns of temperature-responsive genes among different tissues, UpSet analysis was performed for the DEGs identified in females and males, respectively (Figure 3). The results showed that both tissue-specific and shared DEGs were detected across tissues.

In females, the five examined tissues showed distinct DEG overlap patterns (Figure 3a). The largest tissue-specific DEG set was observed in the brain, with 1322 genes detected only in this tissue. The oviduct, liver, and spleen also contained large tissue-specific DEG sets, with 1059, 1025, and 966 genes, respectively. In contrast, the ovary contained a much smaller number of tissue-specific DEGs. Among the shared DEG sets, the largest intersection contained 410 genes shared among the brain, liver, spleen, and oviduct. Several other multi-tissue intersections were also observed, with intersection sizes of 372, 341, 308, and 304 genes. Overall, the female UpSet analysis showed that most temperature-responsive DEGs were either tissue-specific or shared by subsets of tissues rather than uniformly shared by all reproductive and somatic tissues.

In males, the four examined tissues also showed both tissue-specific and shared DEG sets (Figure 3b). The largest tissue-specific DEG set was detected in the liver, with 1392 genes. The brain, testis, and spleen contained 1178, 1010, and 795 tissue-specific DEGs, respectively. The largest shared DEG set contained 376 genes shared among the brain, liver, spleen, and testis. Additional shared DEG sets included 338, 264, 261, 235, 218, 212, 196, 174, 125, and 107 genes, representing different combinations of two or more tissues.

Taken together, the overlap analysis showed that temperature-responsive DEGs were distributed in both tissue-specific and shared patterns. In females, large tissue-specific DEG sets were observed in the brain, oviduct, liver, and spleen, whereas the ovary contributed fewer DEGs to the overlap structure. In males, all four tissues contained large tissue-specific DEG sets, and 376 DEGs were shared across all examined male tissues.

### 3.4. Volcano Plot Analysis of Temperature-Responsive DEGs Across Multiple Tissues

**Figure 4:**
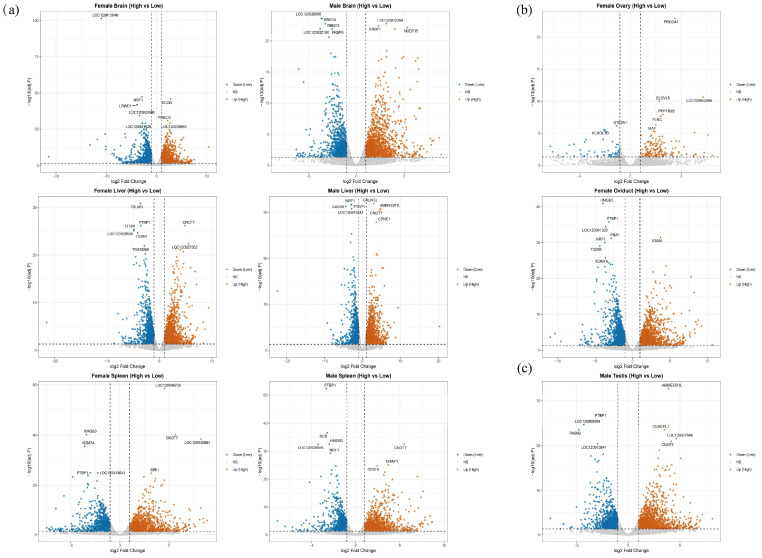
Representative volcano plots of temperature-responsive differentially expressed genes (DEGs) in different tissues of female and male *R. dybowskii.* (**a**) Brain, liver, and spleen in females and males; (**b**) ovary and oviduct in females; (**c**) testis in males. Differential expression was determined by comparing the 12 °C group with the 2 °C group within each tissue using DESeq2. DEGs were defined using the criteria of adjusted *p* value < 0.05 and |log2FoldChange| ≥ 1. Orange dots indicate genes significantly upregulated in the 12 °C group, blue dots indicate genes significantly downregulated in the 12 °C group, and gray dots indicate genes without significant differential expression. The vertical dashed lines represent log2FoldChange = ±1, and the horizontal dashed line represents adjusted *p* value = 0.05. Representative DEGs with relatively large fold changes or high statistical significance are labeled.

To visualize the gene-level distribution of temperature-responsive differentially expressed genes (DEGs), volcano plots were generated for the 12 °C versus 2 °C comparison in representative somatic and reproductive tissues of female and male *R. dybowskii* (Figure 4). DEGs were defined using the criteria of adjusted *p* value < 0.05 and |log2FoldChange| ≥ 1. Orange points indicate genes significantly upregulated in the 12 °C group, blue points indicate genes significantly downregulated in the 12 °C group, and gray points indicate genes without significant differential expression.

Consistent with the DEG statistics summarized in Table 1, the brain, liver, and spleen of both females and males showed broad distributions of significant DEGs on both sides of the volcano plots (Figure 4a). Several representative genes with relatively large fold changes or high statistical significance were labeled. Based on their annotations or known biological functions, these genes could be broadly classified into several functional categories. For example, *SRSF1*, *PTBP1*, and *CNOT7* are associated with RNA processing, RNA binding, splicing regulation, or mRNA turnover; *NRF1* is related to transcriptional regulation and mitochondrial-associated gene expression; PRKCG is involved in signal transduction; and *NUDT15* is associated with nucleotide metabolism. These genes represent highly responsive DEGs in somatic tissues under short-term temperature exposure.

In female reproductive tissues, the ovary and oviduct showed distinct volcano plot patterns (Figure 4b). The ovary displayed a relatively sparse distribution of significant DEGs, whereas the oviduct showed a broader distribution of both upregulated and downregulated genes. In the ovary, representative labeled genes included *PROCA1*, *ELOVL5*, *PPP1R9B*, *FLNC*, and *MAF*. Among these genes, *ELOVL5* is associated with fatty acid elongation and lipid metabolism, while *PPP1R9B*, *FLNC*, and *MAF* are related to cytoskeletal organization, signaling regulation, or transcriptional regulation. In the oviduct, labeled DEGs included *HMGB3*, *PTBP1, PIM3*, *STAT1*, *NRF1*, *TICRR*, and *KDM7A*. These genes were mainly associated with RNA processing, chromatin regulation, signal transduction, transcriptional regulation, cell cycle progression, or DNA replication-related processes.

In the male reproductive tissue, the testis also showed a broad distribution of significant DEGs under the 12 °C versus 2 °C comparison (Figure 4c). Representative labeled genes included *AMMECR1L*, *OSGEPL1*, *CNOT7*, *RAB6B*, and several annotated LOC genes. Functionally, *CNOT7* is related to mRNA turnover, *RAB6B* is associated with intracellular vesicle trafficking, and *OSGEPL1* and *AMMECR1L* may be linked to cellular metabolism or mitochondrial-associated processes based on their annotations. Overall, the volcano plots provided a gene-level visualization of tissue-specific DEG patterns and highlighted representative genes associated with RNA processing, lipid metabolism, signal transduction, transcriptional regulation, cell cycle- or DNA-related processes, and cellular metabolism.

### 3.5. Functional Enrichment Analysis Reveals Metabolic Reprogramming in the Liver Under Thermal Stress

**Figure 5:**
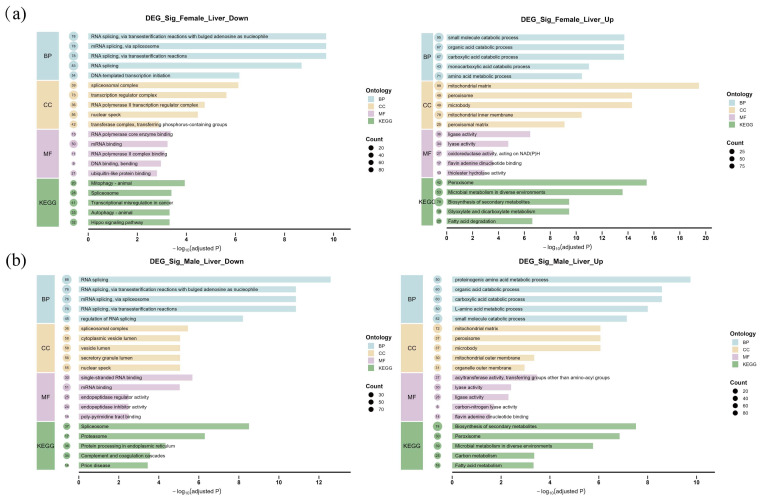
GO and KEGG enrichment analyses of temperature-responsive differentially expressed genes (DEGs) in representative tissues. (**a**) Enriched GO terms and KEGG pathways for upregulated and downregulated DEGs in the liver of female and male *R. dybowskii*; (**b**) enriched GO terms and KEGG pathways for upregulated and downregulated DEGs in the male liver. The abbreviations BP, CC, and MF represent Biological Process, Cellular Component, and Molecular Function, respectively, within the GO categories. KEGG represents the Kyoto Encyclopedia of Genes and Genomes pathway database. Bar length indicates enrichment significance as −log_10_(adjusted *p*), and dot size represents the number of enriched genes.

To further elucidate the functional responses of somatic tissues to thermal stress, we selected the liver as a representative tissue and performed GO and KEGG enrichment analyses separately for the upregulated and downregulated genes in females and males under high-temperature treatment (Figure 5). The results showed that the enrichment patterns were largely consistent between female and male livers, both displaying clear features of metabolic reprogramming.

In both female and male livers, genes upregulated under high-temperature treatment were mainly enriched in biological processes such as small molecule catabolic process, organic acid catabolic process, carboxylic acid catabolic process, amino acid metabolic process, and fatty acid degradation. These genes were also significantly associated with cellular components including the mitochondrial matrix, peroxisome, microbody, and mitochondrial inner membrane, and were enriched in KEGG pathways such as peroxisome, carbon metabolism, biosynthesis of secondary metabolites, and microbial metabolism in diverse environments. These findings indicate that high-temperature treatment activated functional programs related to energy metabolism, lipid utilization, and redox homeostasis in the liver, suggesting that the liver serves as an important metabolic regulatory center in *R. dybowskii* in response to temperature variation. In contrast, genes downregulated under high-temperature treatment in both female and male livers were mainly enriched in GO terms related to RNA splicing, mRNA splicing via spliceosome, RNA splicing via transesterification reactions, spliceosomal complex, and transcription regulator complex, and were significantly enriched in the KEGG pathway spliceosome. These results suggest that certain RNA processing and transcriptional regulatory processes were suppressed or reconfigured under high-temperature conditions.

Overall, the liver exhibited a functional response pattern under thermal stress characterized primarily by enhanced catabolic metabolism and remodeling of RNA regulatory processes, which is highly consistent with the pronounced transcriptional reprogramming observed in the previous differential expression analysis.

### 3.6. Distinct Functional Responses of Reproductive Tissues to Temperature Change

**Figure 6:**
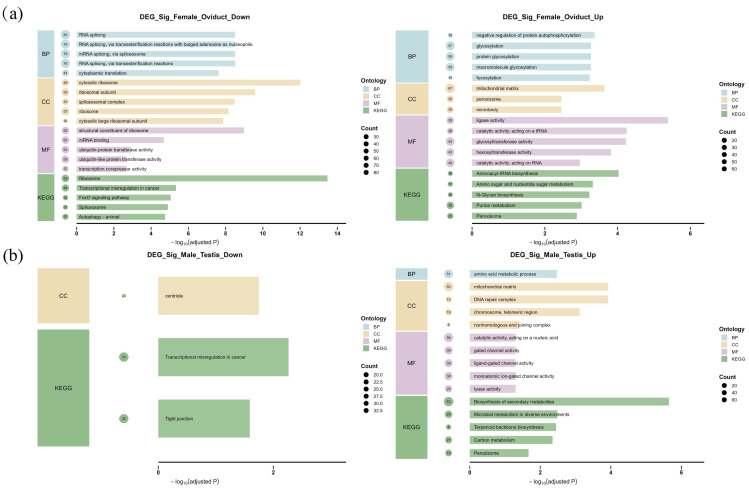
GO and KEGG enrichment analyses of temperature-responsive differentially expressed genes in reproductive tissues of the brown frog (*R. dybowskii*). (**a**) GO and KEGG enrichment results for downregulated genes (**left**) and upregulated genes (**right**) in the female oviduct under high- versus low-temperature conditions. (**b**) GO and KEGG enrichment results for downregulated genes (**left**) and upregulated genes (**right**) in the male testis under high- versus low-temperature conditions. The *x*-axis represents enrichment significance as −log_10_(adjusted *p*). Colors indicate ontology categories (BP, CC, MF, and KEGG), and dot size indicates gene count.

To further elucidate the molecular response characteristics of the reproductive system to temperature change, we performed GO and KEGG enrichment analyses separately on the upregulated and downregulated differentially expressed genes in the female oviduct and male testis (Figure 6). The results revealed clear differences in the functional response patterns of these two reproductive tissues, suggesting pronounced organ-specific differentiation and sex-biased regulation in the reproductive system of *R. dybowskii* under thermal stress.

In the female oviduct, genes downregulated under high-temperature treatment were mainly enriched in terms such as RNA splicing, cytoplasmic translation, ribosome, ribosomal subunit, and structural constituent of ribosome, and were significantly enriched in the KEGG pathways ribosome and spliceosome. These findings indicate that high temperature may suppress ribosome-related translational activity and RNA processing in the oviduct. In contrast, genes upregulated under high-temperature treatment were mainly enriched in glycosylation, protein glycosylation, macromolecule glycosylation, fucosylation, and pathways such as aminoacyl-tRNA biosynthesis, amino sugar and nucleotide sugar metabolism, N-glycan biosynthesis, purine metabolism, and peroxisome, suggesting that the oviduct enhances glycosylation modification, macromolecule processing, and specific metabolic activities under high-temperature conditions, thereby exhibiting marked functional remodeling. In the male testis, genes upregulated under high-temperature treatment were mainly enriched in GO terms such as amino acid metabolic process, mitochondrial matrix, DNA repair complex, chromosome, telomeric region, and nonhomologous end joining complex, and were enriched in KEGG pathways including biosynthesis of secondary metabolites, carbon metabolism, and peroxisome. These results suggest that high-temperature treatment may promote metabolic regulation, mitochondria-related functions, and DNA damage repair activity in the testis, thereby helping to alleviate cellular stress caused by thermal challenge. By contrast, relatively few terms were enriched among the downregulated genes in the testis, mainly involving structure- and junction-related categories such as centriole and tight junction, implying that high temperature may disturb cellular structural stability and the maintenance of the spermatogenic microenvironment in the testis.

Taken together, both the female oviduct and male testis exhibited pronounced responses to thermal stress, but their functional orientations were clearly different: the oviduct was characterized more by the remodeling of glycosylation and secretion-related processing, whereas the testis showed a stronger tendency toward metabolic activation, DNA repair, and structural homeostasis disturbance. Combined with the relatively stable transcriptional state observed in the ovary in the previous analyses, these findings indicate that the reproductive system of *R. dybowskii* does not respond uniformly to temperature change, but instead exhibits clear organ-specific division of labor and sex-biased regulation.

## 4. Discussion

This study was conducted within a multifactorial transcriptomic framework integrating temperature, sex, and tissue, and systematically compared the molecular responses of the brown frog (*R. dybowskii*) across multiple tissues under 2 °C and 12 °C conditions. Overall, the results showed that the response of *R. dybowskii* to temperature variation is not a uniform, whole-body process, but rather exhibits pronounced tissue dependence and sex-biased patterns in the reproductive system. Global PCA and sample correlation analyses demonstrated that tissue type was the primary factor shaping the overall transcriptomic landscape, whereas the effects of temperature and sex were mainly reflected in expression remodeling within individual tissues. This pattern is consistent with the typical response of ectothermic vertebrates to temperature change, in which different tissues often activate distinct adaptive programs according to their specific functional demands. Previous studies in frogs have likewise shown that thermal stress can induce marked tissue-specific oxidative stress, transcriptional remodeling, and metabolic compensatory responses [23,24].

Intersection analysis further demonstrated that the temperature response of *R. dybowskii* follows a dual-layered structure consisting of “shared stress-response modules plus tissue-specific regulation.” On the one hand, a certain number of shared differentially expressed genes were identified across multiple tissues, suggesting that the organism mobilizes a set of conserved basal response programs when facing temperature change. On the other hand, the presence of a large number of tissue-specific DEGs indicates that individual organs differentially process thermal signals according to their own physiological roles. This pattern is particularly important in amphibians, because temperature variation can simultaneously affect energy supply, neural regulation, immune homeostasis, and reproductive function, requiring adaptation to be achieved through the coordinated action of multiple tissues rather than by any single organ alone [25,26]. Similar hierarchical adaptive patterns have also been reported in thermal stress studies of other amphibian species.

Among somatic tissues, the liver was one of the most responsive tissues to temperature change and showed the clearest functional directionality. In the high-temperature group, upregulated genes in the liver were mainly enriched in the catabolism of small molecules, organic acids, carboxylic acids, amino acids, and fatty acids, as well as in terms related to the mitochondrial matrix, peroxisomes, and microbodies. In contrast, downregulated genes were primarily enriched in processes such as RNA splicing, spliceosome, and transcription regulator complex. These findings indicate that the liver underwent pronounced metabolic reprogramming under elevated temperature conditions [27]. On the one hand, it enhanced catabolic metabolism, lipid utilization, and mitochondria-related energy conversion to meet the metabolic demands imposed by thermal stress; on the other hand, it reconfigured RNA processing and transcriptional regulatory processes to optimize resource allocation. Previous studies have shown that the liver of the giant spiny frog (*Quasipaa spinosa*) also exhibits increased reactive oxygen species (ROS) levels, altered antioxidant enzyme activities, and stress-related transcriptional remodeling under cold and heat stress [28]. Likewise, transcriptomic analyses of the liver in the high-altitude frog *Nanorana pleskei* have revealed features of metabolic compensation and energy maintenance under high-temperature conditions, which are highly consistent with the pattern of “metabolic enhancement plus regulatory remodeling” observed in the present study [24]. The supplementary results showed that the brain is also an important tissue involved in the temperature response of *R. dybowskii*. In the female brain, downregulated genes were enriched in terms such as neuropeptide signaling pathway, regulation of hormone secretion, and receptor ligand activity, whereas upregulated genes were mainly associated with tRNA metabolism, DNA repair, protein methylation, mitochondrial matrix, and aminoacyl-tRNA biosynthesis. A similar trend was observed in the male brain, where upregulated genes were primarily related to mitochondrial gene expression, mitochondrial translation, and DNA repair, while downregulated genes were more closely associated with processes such as chromatin organization, protein kinase complex, and adherens junction. Taken together, the brain response to temperature change appears to involve not merely a general cellular stress reaction, but more likely the remodeling of neuroendocrine regulation and signal transduction, together with enhanced mitochondrial function and nucleic acid repair [29]. Previous studies have shown that acute heat exposure can induce marked oxidative damage and changes in antioxidant defense in frog brain tissue, indicating that the central nervous system is a key locus of temperature sensitivity in ectothermic animals [30,31]. Against this background, the enrichment of terms related to neuropeptides, hormonal regulation, mitochondria, and DNA repair in the present study likely reflects the involvement of the central regulatory system in coordinating systemic adaptive responses to temperature change in *R. dybowskii*.

The Supplementary Materials results for the spleen further indicate that temperature change affects not only metabolism and the nervous system, but also has a pronounced impact on immune-related tissues. In the female spleen, downregulated genes were enriched in cytoplasmic translation, protein targeting to ER, ribosomal subunit, and structural constituent of ribosome, and were also significantly enriched in the KEGG pathway ribosome. In contrast, upregulated genes were mainly associated with tRNA metabolism, amino acid activation, mitochondrial matrix, peroxisome, DNA replication, and aminoacyl-tRNA biosynthesis. The male spleen exhibited a somewhat different regulatory pattern: its downregulated genes were primarily related to RNA splicing, spliceosome, histone acetyltransferase complex, transcription corepressor activity, and the JAK–STAT signaling pathway, whereas its upregulated genes were likewise concentrated in categories associated with tRNA metabolism, amino acid metabolism, mitochondria, peroxisomes, and DNA replication. Overall, the spleen under elevated temperature was characterized by a reduction in translation- and transcription-related processes, accompanied by enhanced metabolic activity, replication, and aminoacylation-related processes. Recent research on the wood frog (*Lithobates sylvaticus*) has shown that splenic immune gene expression is highly sensitive to seasonal transitions between cold and warm conditions, with seasonal dynamics that are even stronger than those in some other tissues, which is consistent with the pronounced transcriptional remodeling observed in the spleen in the present study [32,33,34]. These findings suggest that the spleen of *R. dybowskii* may play an important role in immune resource reallocation and stress-defense regulation under temperature change.

Overall, the present study supports a clearly tissue-dependent model of temperature response in *R. dybowskii*: the liver primarily functions in metabolic buffering and energy reprogramming, the brain likely contributes to neuroendocrine integration and the regulation of cellular repair, the spleen is involved in the reallocation of translational and transcriptional resources as well as the remodeling of immune homeostasis, and the reproductive system further exhibits a multilayered structure characterized by ovarian homeostasis maintenance, oviductal remodeling of secretory and processing functions, and metabolic/repair activation in the testis. For the brown frog, which inhabits cold-temperate environments over the long term, this multi-tissue and multi-level functional division may represent a key adaptive mechanism that enables the species to balance survival and reproduction under seasonal temperature fluctuations. However, it should be noted that the present study was based on two temperature treatments and a single short-term exposure time point. Therefore, the observed transcriptomic differences should be interpreted as acute or short-term molecular responses to temperature exposure rather than direct evidence of long-term thermal adaptation. In addition, this study did not include biochemical measurements, qPCR validation, or functional assays of candidate genes. Future studies integrating time-course experiments, long-term acclimation treatments, physiological and biochemical parameters, and experimental validation of key genes will be necessary to determine whether these transcriptional changes contribute to adaptive thermal regulation in *R. dybowskii*.

## 5. Conclusions

Based on multi-tissue transcriptomic analysis, this study systematically revealed the molecular response patterns of the brown frog (*R. dybowskii*) under 2 °C and 12 °C conditions. The results showed that the transcriptomic response of *R. dybowskii* to temperature variation is highly tissue-specific, with the overall expression pattern being primarily driven by tissue type, whereas temperature effects are mainly reflected in functional remodeling within individual tissues. Among these, the liver was characterized mainly by metabolic reprogramming, while the brain and spleen exhibited responses associated with neural regulation and immune homeostasis, respectively. The reproductive system displayed more pronounced organ-specific division of labor and sex-biased patterns: the ovary remained relatively stable, the oviduct mainly underwent remodeling related to secretory processing and glycosylation, and the testis showed enhanced metabolism, activation of DNA repair, and disturbance of structural homeostasis. Collectively, adaptation of *R. dybowskii* to temperature change is not a uniform whole-body response, but rather depends on coordinated regulation among multiple tissues and differential functional specialization within the reproductive system. This study provides new transcriptomic evidence for understanding the molecular mechanisms of temperature response in amphibians and the regulation of reproductive physiology in *R. dybowskii*.

## Figures and Tables

**Table 1:** Summary of temperature-responsive differentially expressed genes in different tissues of female and male *R. dybowskii*.

Tissue	Gender	Up	Down	Total
Liver	Female	1507	1389	2896
Liver	Male	1623	1303	2926
Brain	Female	1495	1305	2800
Brain	Male	1578	1142	2720
Spleen	Female	1516	1440	2956
Spleen	Male	1435	1125	2560
Ovary	Female	167	59	226
Oviduct	Female	1617	1460	3077
Testis	Male	1493	1125	2618

## Data Availability

The raw sequencing data generated during the current study have been deposited in the Genome Sequence Archive (GSA) at the National Genomics Data Center (NGDC), China National Center for Bioinformation (CNCB). The RNA-seq datasets for female brown frogs (*Rana dybowskii*) are publicly accessible under the accession number CRA012717, and the datasets for male brown frogs are accessible under the accession number CRA042544. The data can be accessed via the permanent URL: https://ngdc.cncb.ac.cn/gsa/ (accessed on 5 March 2026).

## Supplementary Materials

The following supporting information can be downloaded at: https://www.mdpi.com/article/10.3390/cimb48060583/s1.
