# Supplementary material for: Multi-Tissue Transcriptomic Analysis Reveals Tissue-Specific Thermal Responses and Sex-Biased Functional Differentiation in Reproductive Tissues of the Brown Frog (Rana dybowskii)
Source: Curr Issues Mol Biol. 2026 Jun 1;48(6):583. doi: 10.3390/cimb48060583 (PMC13297849; doi:10.3390/cimb48060583)
Supplement: Supplementary file 1 [file cimb-48-00583-s001.zip › cimb-4309337-supplementary.pdf]

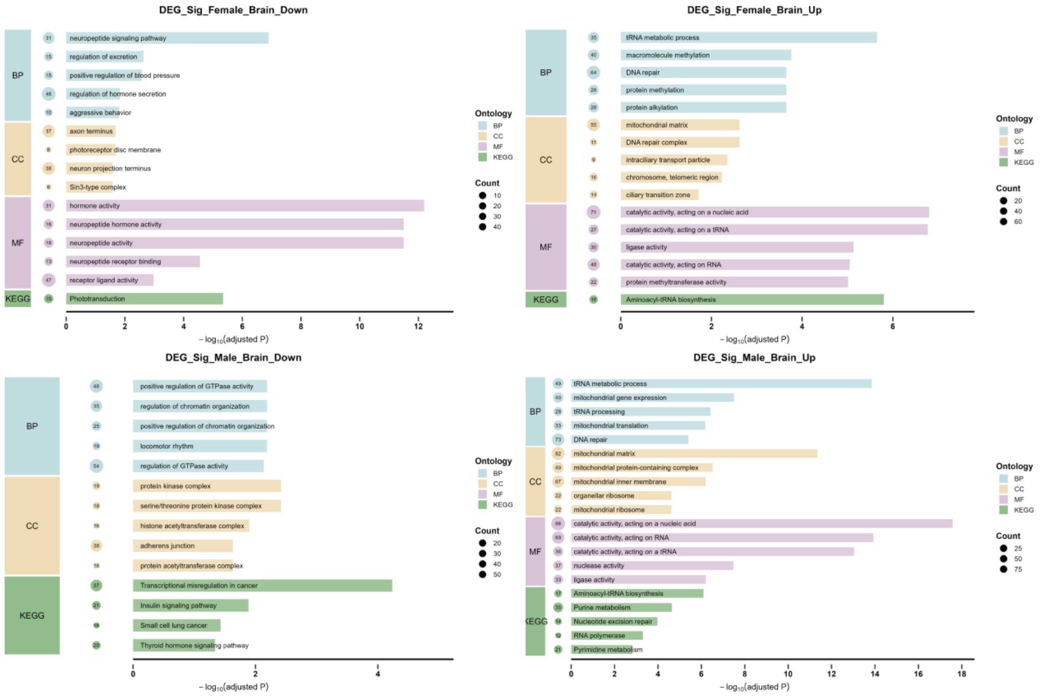

Figure S1. GO and KEGG enrichment analysis of differentially expressed genes in the brain of the brown frog (*Rana dybowskii*). GO and KEGG enrichment results for differentially expressed genes in the brain of female and male brown frogs under high- versus low-temperature conditions. The upper left panel shows downregulated genes in female brain (DEG_Sig_Female_Brain_Down), the upper right panel shows upregulated genes in female brain (DEG_Sig_Female_Brain_Up), the lower left panel shows downregulated genes in male brain (DEG_Sig_Male_Brain_Down), and the lower right panel shows upregulated genes in male brain (DEG_Sig_Male_Brain_Up). The x-axis represents enrichment significance as -$\log_{10}$(adjusted *P*). Colors indicate ontology categories (BP, CC, MF, and KEGG), and dot size indicates gene count.

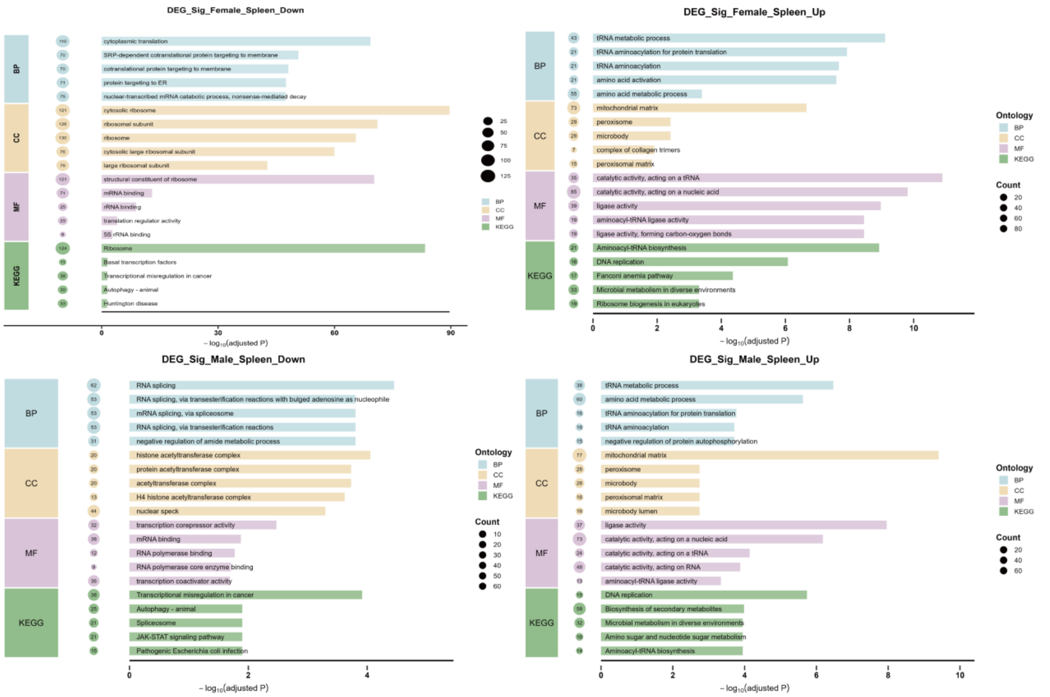

Figure S2. GO and KEGG enrichment analysis of differentially expressed genes in the spleen of the brown frog (*Rana dybowskii*). GO and KEGG enrichment results for differentially expressed genes in the spleen of female and male brown frogs under high- versus low-temperature conditions. The upper left panel shows downregulated genes in female spleen (DEG_Sig_Female_Spleen_Down), the upper right panel shows upregulated genes in female spleen (DEG_Sig_Female_Spleen_Up), the lower left panel shows downregulated genes in male spleen (DEG_Sig_Male_Spleen_Down), and the lower right panel shows upregulated genes in male spleen (DEG_Sig_Male_Spleen_Up). The x-axis represents enrichment significance as -$\log_{10}$(adjusted *P*). Colors indicate ontology categories (BP, CC, MF, and KEGG), and dot size indicates gene count.
